# *Caenorhabditis elegans* strain sensitivity to sodium arsenite exposure is varied based on age and outcome measured

**DOI:** 10.17912/micropub.biology.000186

**Published:** 2019-10-22

**Authors:** Kathleen A Hershberger, Tess C Leuthner, Tanner A Waters, Joel N Meyer

**Affiliations:** 1 Nicholas School of the Environment, Duke University, Durham, NC 27708; 2 Institute of the Environment and Sustainability, University of California, Los Angeles (UCLA), Los Angeles, CA 90095

**Figure 1. Sensitivity of electron transport chain mutants to sodium arsenite exposure:**
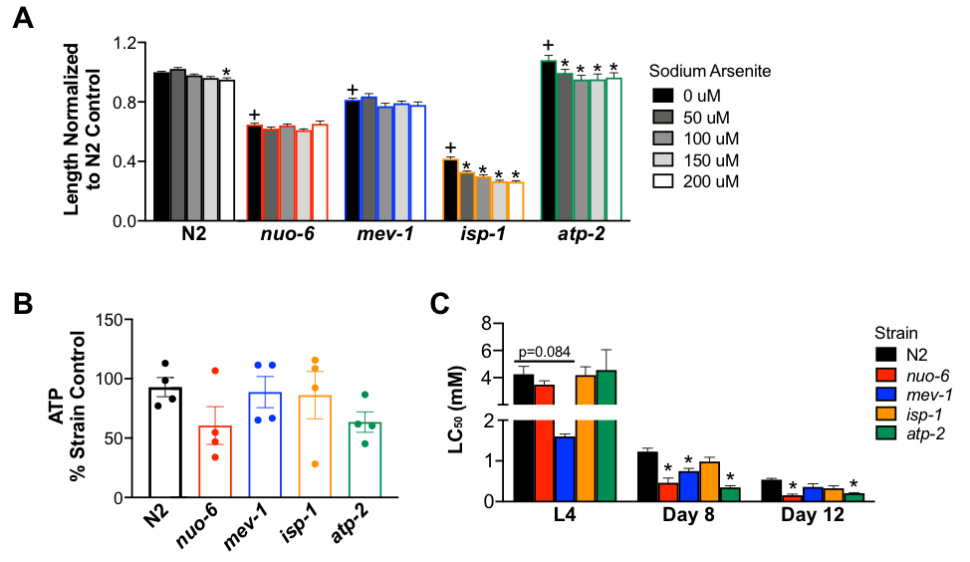
A) Length of electron transport chain *C. elegans* mutant strains was determined after a 72-hour exposure of synchronized L1 stage nematodes to various concentrations of sodium arsenite. Bars represent average length normalized to the N2 control from 2 independent experiments with n=9-10 worms per exposure concentration per experiment. Two-way ANOVA with Bonferroni test to account for multiple comparisons. B) ATP was measured and normalized to protein per worm immediately following a 48-hour exposure (beginning at L3 stage) to 500 μM sodium arsenite. Each circle represents one independent experiment completed with three technical replicates. C) The average LC_50_ was calculated after L4, 8-day old, and 12-day old nematodes were exposed to sodium arsenite for 24 hours. Bars represent average LC_50_ of three independently generated dose response curves. Error bars represent standard error. One-way ANOVA per age group, Dunnett’s test to account for multiple comparisons. *p ≤ 0.05 compared to strain control; + p ≤ 0.05 compared to N2 control.

## Description

Arsenic is a toxicant with multiple intracellular targets, including many mitochondrial enzymes. Previous work in *C. elegans* has shown that strains deficient in complexes I, II, and III (but not complex V) have increased sensitivity to sodium arsenite, suggesting that the electron transport chain is a key target of arsenic toxicity (Luz *et al.* 2016). However, in that work, the only outcome used to determine strain sensitivity to arsenic was larval growth. Based on these results, we used complex mutants (*nuo-6* (complex I), *mev-1* (complex II), *isp-1* (complex III), and *atp-2* (complex V)) to further explore the physiological effects of sodium arsenite induced electron transport chain dysfunction. We used three different assays to ask the same question: which electron transport chain mutant strain is most sensitive to sodium arsenite? Interestingly, we found that strain sensitivity to sodium arsenite depends on the outcome measured. These data indicate that single assays may not provide a complete picture of nematode response to an exposure and suggests that complete characterization of nematode response requires analyzing multiple endpoints.

Nematodes were synchronized as L1 larvae by standard egg prep methods. For all experiments, biological replicates were performed with independent egg preps on different days. To minimize the potential for different bleaching events to affect outcomes and confound the strain-specific differences we sought to characterize, strains compared in a given experiment were bleached at the same time, or mutant strain data was normalized to N2s bleached at the same time (exceptions to this statement are the lethality curves generated for *nuo-6* and *isp-1* mutants at the L4 stage which were performed in isolation on separate days) (Lewis and Felming 1995). First, synchronized nematodes were treated with sodium arsenite in liquid K-medium at concentrations from 0-200 μM for 72 hours at 20 °C. Worm length was measured following this exposure using the NIS-Elements BR 3.2 program (Bodhicharla *et al.* 2014) (Fig. 1A). The concentrations of sodium arsenite used in this assay were lower than those previously reported (Luz *et al.* 2016) and yielded slightly different results. We found that *isp-1* and *atp-2* strains had decreased worm length when exposed to low concentrations of sodium arsenite, but that length of the *nuo-6* and *mev-1* strains were not affected. Next, we measured ATP in worms following sodium arsenite exposure (Fig. 1B). Synchronized worms were exposed to 500 μM sodium arsenite beginning 2 days post hatch for 48 hours. Immediately after the exposure, steady state ATP levels were measured using the Promega CellTiterGlo Luminescent assay and normalized to protein per worm (adapted from Bailey *et al.* 2016). Although we report no significant differences, there is a strong trend of decreased ATP in the *nuo-6* and *atp-2* mutant strains. Finally, the 24 hour LC_50_ to sodium arsenite was determined in the mutant strains at different life stages. Strains were exposed to various concentrations of sodium arsenite for 24 hours at the L4 stage, 8 days of age, and 12 days of age. 10 worms per well (three wells per concentration) were exposed and wells were scored immediately after the exposure to determine percent alive in each well. At the L4 stage, only the *mev-1* mutant strain appeared to have altered sensitivity to sodium arsenite. However, in the 8-day old worms, the *nuo-6*, *mev-1*, and *atp-2* strains were more sensitive to sodium arsenite compared to the N2 strain. This was also observed in the 12-day old worms with the exception that the *mev-1* strain did not show increased sensitivity to arsenic.

Together, our data shows that strain sensitivity to arsenic is dependent on the outcome measured. For example, the *isp-1* strain has decreased growth when exposed to low concentrations of sodium arsenite but does not appear to have differences in ATP content or lethality compared to N2 controls. In contrast, the *nuo-6* strain showed no difference in growth compared to N2 controls during sodium arsenite exposure but appeared to have decreased ATP and had a significantly decreased LC_50_ at 8 and 12 days of life. Additionally, we show that even within the same assay, strains responded differently to sodium arsenite exposure at different life stages. While *mev-1* appeared to be most sensitive to sodium arsenite at the L4 stage, the *mev-1* strain did not show an increased sensitivity to sodium arsenite in the 12-day old worms. Three separate assays with different exposure concentrations, age of worm, and outcome measured yielded different results to our original research question. This result highlights the importance in toxicology of not just genetic background in modulating sensitivity, but also age—i.e., gene × environment × age interactions. The mechanisms responsible for these differences remain to be determined but may include toxicokinetic (e.g., cuticular and xenobiotic defense mechanisms) as well as toxicodynamic (e.g., differential reliance of specific mitochondrial metabolic processes at different life stages) differences. This work reveals how assay design influences answers to scientific questions, and highlights the importance of measuring multiple outcomes to characterize nematode strains.

## Reagents

Strains

Wild type (N2 Bristol)

ETC Complex I: *nuo-6*(*qm200*), CGC Strain MQ1333

ETC Complex II: *mev-1*(*kn1*), CGC Strain TK22

ETC Complex III: *isp-1*(*qm150*), CGC Strain MQ887

ETC Complex V: *atp-2*(*ua2*), CGC Strain LB127
